# Characteristics and temporal trends of mortality rates in children and adolescents in Mato Grosso and Brazil, 2009-2020

**DOI:** 10.1590/S2237-96222022000300017

**Published:** 2022-12-02

**Authors:** Mônia Maia de Lima, Alexsandra Rodrigues de Mendonça Favacho, Reinaldo Souza-Santos, Silvana Granado Nogueira da Gama

**Affiliations:** 1 Fundação Oswaldo Cruz, Programa de Pós-Graduação em Epidemiologia, Equidade e Saúde Pública, Campo Grande, MS, Brazil; 2 Escola Nacional de Saúde Pública, Departamento de Endemias Samuel Pessoa, Rio de Janeiro, RJ, Brazil; 3 Escola Nacional de Saúde Pública, Departamento de Epidemiologia e Métodos Quantitativos em Saúde, Rio de Janeiro, RJ, Brazil

**Keywords:** Mortality, Time Series Studies, Causes of Death, External Causes, Transport Accidents

## Abstract

**Objective:**

to analyze the characteristics and temporal trend of mortality rates in the population aged 5 to 14 years in Mato Grosso state and in Brazil, from 2009 to 2020.

**Methods:**

this was an ecological time-series study, based on data taken from the Mortality Information System. Descriptive and trend analyses were performed, using the joinpoint regression model and calculating the average annual percentage change (AAPC).

**Results:**

in Brazil and in Mato Grosso state, deaths were predominantly male, preventable and due to external causes. A falling trend was identified for Brazil (5-9 years AAPC: -2.9; 95%CI -4.3;-1.6 and 10-14 years AAPC: -2.5; 95%CI -3.3;-1.8), while a stationary trend was found in Mato Grosso (5-9 years AAPC: -2.0; 95%CI -5.6;1.7 and 10-14 years AAPC: -0.1; 95%CI -5.9;6.1).

**Conclusion:**

the stable trend of mortality at high levels demands urgent interventions to reduce it.

**Study contributions**

**Main results**

The magnitude of male deaths, preventable deaths and deaths from external causes was high in Mato Grosso state and in Brazil as a whole. In Mato Grosso, the mortality trend in the 5 to 9 and 10 to 14 age groups was stable, while for Brazil as a whole the trend was falling.

**Implications for services**

Morbidity and mortality related to external causes overburden emergency and rehabilitation services. Deaths from these causes, considered preventable, lead to years of potential life lost, with epidemiological, social, and economic consequences.

**Perspectives**

Multifactorial interventions to reduce risk factors are essential. Prevention policies, especially for traffic accidents, cannot be delayed and are capable of provoking important changes in the mortality trend.

## Introduction

The inclusion of mortality among children under 5 years of age, referred to as child mortality, as part of the Millennium Development Goals (MDGs) for the period 1990-2015, and as part of the Sustainable Development Goals (SDGs) to be met by 2030, points to its relevance as an indicator of the population’s health population. Monitoring child mortality guides the development of strategies to reduce illness and death in this age group.^1^

In addition to the efforts directed towards reducing the child mortality rate, mortality in the population between 5 and 14 years of age also deserves to be highlighted, since it includes deaths that are for the greater part preventable.^2^ ^,^ ^3^ ^,^ ^4^ Between 1990 and 2016, the risk of death for those in the 5-14 age group fell by 51% worldwide, although the trend slowed in the 2000s. During this period, the reduction in deaths of those aged 5-9 was greater than that among those in the 10-14 age group.^3^

External causes, especially road traffic accidents, were the leading cause of deaths in the 5-14 age group in Europe from 1990 to 2016.^5^ In low- and middle-income countries, besides external causes, cancer, respiratory, neurological and infectious diseases have stood out as causes of death in this age group.^2^

A study conducted in India, China, Mexico and Brazil, which analyzed characteristics and trends in mortality in those aged 5-14 between 2005 and 2016, showed similarities of these deaths in terms of the underlying basic causes and the proportion of deaths due to ill-defined causes. However, variations were found between the order and proportion of causes of death, the 5-9 age group compared to the 10-14 age group, as well as between the sexes and the countries included in the study.^2^

Between 2009 and 2020, 867,548 Brazilians under the age of 20 lost their lives. Despite accounting for some 12% of all deaths in the population, the 5-14 age group corresponds to around 100,000 deaths, mostly classified as preventable, according to the Brazilian National Health System (*Sistema Único de Saúde do Brasil* - SUS) List of Causes of Deaths Preventable through Interventions (5-74 age group).^6^ ^,^ ^7^

Still in relation to the period between 2009 and 2020, analysis of mortality rates for 5-9 years old and 10-14 years old in Brazil indicates that the Northern region led the national ranking, with higher values (357.1/100,000 inhab. - 5-9 years; 432.9/100,000 inhab. - 10-14 years), followed by the Midwest region (298.0/100,000 inhab. - 5-9 years; 394.8/100,000 inhab. - 10-14 years). The state of Mato Grosso, located in the Midwest region, had the highest values in that region in both age groups (321.9/100,000 inhab. - 5-9 years; 430.0/100,000 inhab. - 10-14 years), surpasssing some states in the Northern region and all the national rates.^6^

In Mato Grosso, around 70% of deaths of people under 20 years of age occurring between 2009 and 2020 were due to preventable causes: accidents, assault and communicable diseases.^6^ Preventable deaths, in general, are the result of failures in the prevention, diagnosis and treatment of conditions that determine their occurrence, as well as reflecting unsatisfactory levels of health and life contexts.^8^ The objective of this study was to analyze the characteristics and temporal trend of mortality rates in the population aged 5 to 14 years in Mato Grosso state and in Brazil, from 2009 to 2020.

## Methods

### Design

This was an ecological time-series study, which used data taken from the Mortality Information System (*Sistema de Informação sobre Mortalidade* - SIM), covering a 12-year period (2009 to 2020), having as its units of analysis both Brazil and the state of Mato Grosso.

### Background

The SIM system aggregates information regarding the characteristics of deaths certified on death certificates throughout the national territory. The database made available by the SUS Department of Information Technology (DATASUS) is freely accessible and enables countless data cross-referencing. Mortality statistics are an important tool in identifying the population’s health problems and for informing public health policy planning and management.^9^ ^,^ ^10^

### Participants

We analyzed the data on the deaths of children and adolescents aged 5 to 14 years old living in both the state of Mato Grosso and also in Brazil as a whole that occurred between 2009 and 2020. 

### Variables

Apart from the “death” outcome, we extracted the aggregate data on the following variables:

- Sex (male; female);
- Age group (5-9 years; 10-14 years);
- Race/skin color (White; Black; mixed race; Indigenous; Asian; unknown; other);
- Underlying cause of death, according to the most recurrent chapters of the International Statistical Classification on Diseases and Related Health Problems - ICD-10 (Certain infectious and parasitic diseases - A00-B99; Neoplasms - C00-D48; Diseases of the nervous system - G00-G99; Diseases of the respiratory system - J00-J99; External causes - V01-Y98); and
- Classification of the preventability of death, according to groups of causes, as per the SUS List of Causes of Deaths Preventable through Interventions (5-74 age group), the ICD listed description of which can be consulted in Malta et al. (2018), namely:^7^ deaths reducible by vaccination actions; deaths reducible by health promotion, communicable disease prevention, control and adequate care actions; deaths reducible by health promotion, non-communicable disease prevention, control and adequate care actions; deaths reducible by maternal causes prevention, control and adequate care actions; deaths reducible by intersectoral and health promotion, external causes prevention and care actions; ill-defined causes; other causes (not clearly preventable).

### Data collection

The mortality data were extracted from DATASUS, taking the underlying causes of death according to the ICD-10 chapters. We considered the five chapters which occurred most frequently. Our analysis of preventability was based on the updated SUS List of Causes of Deaths Preventable through Interventions (5-74 age group).^7^

The population data were extracted from the website of the Brazilian Institute of Geography and Statistics (*Instituto Brasileiro de Geografia e Estatística* - IBGE), which provides the 2010 Census data as well as population estimates up to the year 2021.

### Data analysis

The data were tabulated using the Health Information Tabulator (*Tabulador de Informações em Saúde* - TabNet) before being exported to Excel^®^ spreadsheets. Deaths were stratified by age group and sex. 

The raw annual mortality rates per 100,000 inhabitants for each age group were calculated both for Brazil as a whole and also for Mato Grosso state, as well as the total rate for the period analyzed. The following formula was used for this calculation:

Mortality rate = (number of deaths/population) x 100,000

We also calculated the standardized rates by age group, using the direct method, taking the reference to be the age structure of the Brazilian population estimated by the IBGE for each year of analysis. Standardization was necessary in order for the mortality rates to be comparable between each other and throughout the period studied. 

After standardization, the mean value for each age group was calculated, as well as the standard deviation and the difference between the rates at the beginning and end of the period.

Descriptive analysis of the mortality data at the study sites was performed. Besides the mortality rates, the absolute frequencies of deaths and their distribution by race/skin color and underlying cause were presented, both for the comparison between Brazil and Mato Grosso, and for comparison between age groups in Mato Grosso. 

When analyzing race/skin color, we grouped together the “Black” and “Indegenous” categories, due to the small number of cases and because they are more vulnerable groups, and we also grouped together the “unknown” and “other” categories.

We performed temporal trend analyses for Brazil as a whole and for Mato Grosso state, comparing the resulting annual percentage change (APC) and average annual percentage change (AAPC).

### Statistical methods

We applied the chi-square test (X^2^), using R software to test the homogeneity of the proportions obtained through the descriptive analysis.

When analyzing the mortality rate temporal trends, we used the JoinPoint Regression Program, version 4.9.1.0, dated April 2022 (Statistical Research and Applications Branch, National Cancer Institute), which, based on the Monte Carlo permutation method, estimated APC and AAPC, taking a 95% confidence interval (95%CI) and a 5% significance level.

The changes in the mortality rate values, both in terms of increase and reduction, are the basis for identifying jointpoints. Following the pattern of the method, the number of jointpoints varies according to the number of points (in this case, years) in the database analyzed. Taking the slope of the regression line, it is possible to identify these points, which allows the temporal trend to be classified as stationary (p-value > 0.05), rising (p-value < 0.05 and positive regression coefficient) or falling (p-value < 0.05 and negative regression coefficient).^11^ ^,^ ^12^ ^,^ ^13^ The jointpoint regression model was applied for both age groups and for the national and state scenarios.

### Ethical aspects

As this study is based on analysis of public domain and free access secondary and grouped data, with no identification of the individuals involved, it received Ethics Waiver Opinion No. 09/2022 from the *Escola Nacional de Saúde Pública* Research Ethics Committee. 

## Results

Between 2009 and 2020, 876 deaths of people aged 5-9 years were recorded, and 1,192 in the 10-14 years age group in Mato Grosso, while in Brazil, there were 42,661 deaths of people aged 5-9 years and 60,323 in the 10-14 years age group. 

Throughout the time series, from 2009 to 2020, the mortality rate in the 10-14 years age group surpassed that of the younger group, both in Mato Grosso (321.9/100,000 inhab. - 5-9 years; 430.0/100,000 inhab. - 10-14 years) and also in Brazil (268.3/100,000 inhab. - 5-9 years; 360.1/100,000 inhab. - 10-14 years). Male mortality exceeded female mortality, both in Mato Grosso and in Brazil (Mato Grosso: 439.9/100,000 inhab. - male and 312.2/100,000 inhab. - female; Brazil: 369.2/100,000 inhab. - male and 259.3/100,000 inhab. - female). The Mato Grosso state rates were higher than the national rates in all strata. 

**Figure 1:**
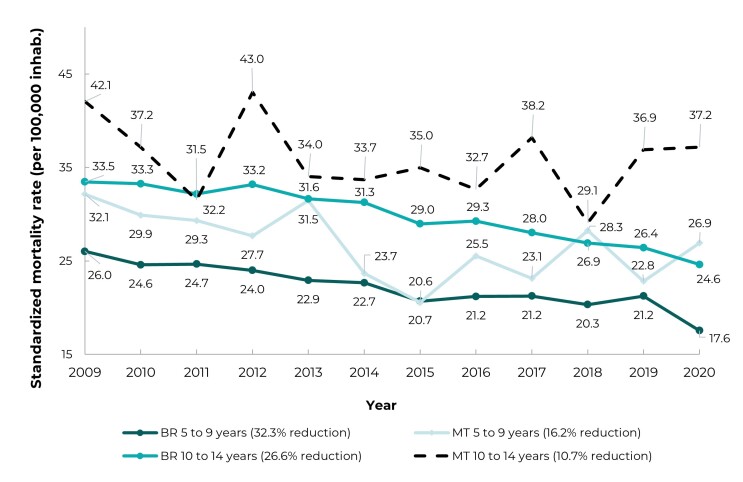
Changes in mortality rates in the 5-9 and 10-14 years age groups, Brazil and Mato Grosso, 2009-2020

There was a reduction in mortality rates at the end of the period analyzed in relation to the beginning, for both age groups, both in Mato Grosso (from 32.1 to 26.9/100,000 inhab., for 5-9 years old; from 42.1 to 37.2/100,000 inhab., for 10-14 years old) and also in Brazil as a whole (from 26.0 to 17.6/100,000 inhab., for 5-9 years old; from 33.5 to 24.6/100,000 inhab., for 10-14 years old). A greater reduction was found in the mortality of children aged 5-9 years in Brazil as a whole (-32.3%), as per Figure 1.

The mortality rates in Mato Grosso exceeded the national rates in both age groups analyzed (average in Mato Grosso: 26.8/100,000 inhab., for 5-9 years old, and 35.9/100,000 inhab. for 10-14 years old; average in Brazil: 22.3/100,000 inhab., for 5-9 years old, and 29.9/100,000 inhab. for 10-14 years old). The exceptions were the year 2015, for children aged 5-9 years, and 2011, for children aged 10-14 years. The values fluctuated more in Mato Grosso in both groups, with greater variability in those aged 10-14 years old (Figure 1). 

In both Brazil and Mato Grosso, there was a predominance of male deaths in both age groups (Mato Grosso: 58.2%, 5-9 years, and 60.6%, 10-14 years; Brazil: 57.2%, 5-9 years, and 61.5%, 10-14 years) (Table 1).

**Table 1:** Number and percentage of deaths by age group (5-9 and 10-14 years), sex, race/skin color, underlying cause and preventable causes, Brazil and Mato Grosso, 2009-2020

Variables	Brazil					Mato Grosso				
	5 to 9 years		10 to 14 years		p-value^a^	5 to 9 years		10 to 14 years		p-value^a^
	n	%	n	%		n	%	n	%	
**Sex**										
Male	24,412	57.2	37,101	61.5	< 0.001	510	58.2	722	60.6	0.282
Female	18,242	42.8	23,216	38.5		366	41.8	470	39.4	
Unknown	7	0.0	6	0.0		-	0.0	-	0.0	
**Race/skin color**										
White	16,763	39.3	21,985	36.4	< 0.001	276	31.5	375	31.5	0.044
Mixed race	20,694	48.5	30,694	50.9		496	56.6	701	58.8	
Black and Indigenous	2,690	6.3	4,505	7.5		79	9.0	102	8.5	
Unknown and other	2,514	5.9	3,139	5.2		25	2.9	14	1.2	
**Underlying cause**										
External causes	12,865	30.2	25,347	42.0	< 0.001	311	35.5	561	47.1	< 0.001
Neoplasms	7,164	16.8	7,678	12.7		108	12.3	136	11.4	
Nervous system diseases	4,932	11.6	5,928	9.8		92	10.5	95	8.0	
Respiratory diseases	3,679	8.6	3,723	6.2		79	9.0	71	5.9	
Infectious diseases	3,084	7.2	3,125	5.2		72	8.2	66	5.5	
Other causes	10,937	25.6	14,522	24.1		214	24.5	263	22.1	
**Preventable causes**										
Total deaths from preventable causes	23,315	54.7	37,788	62.6	< 0.001	519	59.2	783	65.7	< 0.001
Reducible by vaccination actions	49	0.1	80	0.1		-	0.0	3	0.2	
Reducible by actions related to care of communicable diseases	5,292	12.4	5,468	9.1		118	13.5	100	8.4	
Reducible by actions related to care of non-communicable diseases	5,109	12.0	6,724	11.1		90	10.2	114	9.6	
Reducible by actions related to care of maternal causes	-	0.0	196	0.3		-	0.0	5	0.4	
Reducible by actions related to care of external causes	12,865	30.2	25,347	42.0		311	35.5	561	47.1	
Total deaths	42,661		60,323			876		1,192		

a) Chi-square test of independence.

The distribution of deaths according to race/skin color and underlying cause reveals similarities between the state and national scenarios, with a predominance of deaths of mixed race individuals (Mato Grosso: 56.6%, for 5-9 years old, and 58.8% for 10-14 years old; Brazil: 48.5%, for 5-9 years old, and 50.9% for 10-14 years old) and for deaths from external causes (Mato Grosso: 35.5%, for 5-9 years old, and 47.1% for 10-14 years old; Brazil: 30.2%, for 5-9 years old, and 42.0% for 10-14 years old) in relation to other causes, as per Table 1.

Table 1 shows the magnitude of preventable deaths in the age groups (Mato Grosso: 59.2%, for 5-9 years old, and 65.7% for 10-14 years old; Brazil: 54.7%, for 5-9 years old, and 62.6% for 10-14 years old), especially deaths that can be reduced by actions related to care of external causes. 

In Mato Grosso, the distribution of deaths by the main groups of external causes was similar in both age groups for females, with a predominance of transport accidents (55.8%, for 5-9 years old, and 48.1% for 10-14 years old). Among males, despite the predominance of transport accidents in both age groups, the proportion of deaths from assaults among individuals aged 10-14 years old (20.4%) stood out (Table 2).

**Table 2:** Distribution of deaths from external causes among residents, by age group, sex and main ICD-10^a^ groups, Mato Grosso, 2009-2020

Groups of underlying causes of death from external causes	Sex					
	Male			Female		
	Age group (in years)			Age group (in years)		
	5 to 9	10 to 14	p-value^b^	5 to 9	10 to 14	p-value^b^
	n (%)	n (%)		n (%)	n (%)	
Transport accidents	87 (45.5)	150 (40.3)	0.001	67 (55.8)	91 (48.2)	0.095
Drowning and submersion	49 (25.7)	70 (18.9)		26 (21.7)	32 (16.9)	
Assault	14 (7.3)	76 (20.4)		10 (8.3)	32 (16.9)	
Other external causes	41 (21.5)	76 (20.4)		17 (14.2)	34 (18.0)	

a) International Statistical Classification of Diseases and Related Health Problems - ICD-10; b) Chi-square test of independence.

**Figure 2:**
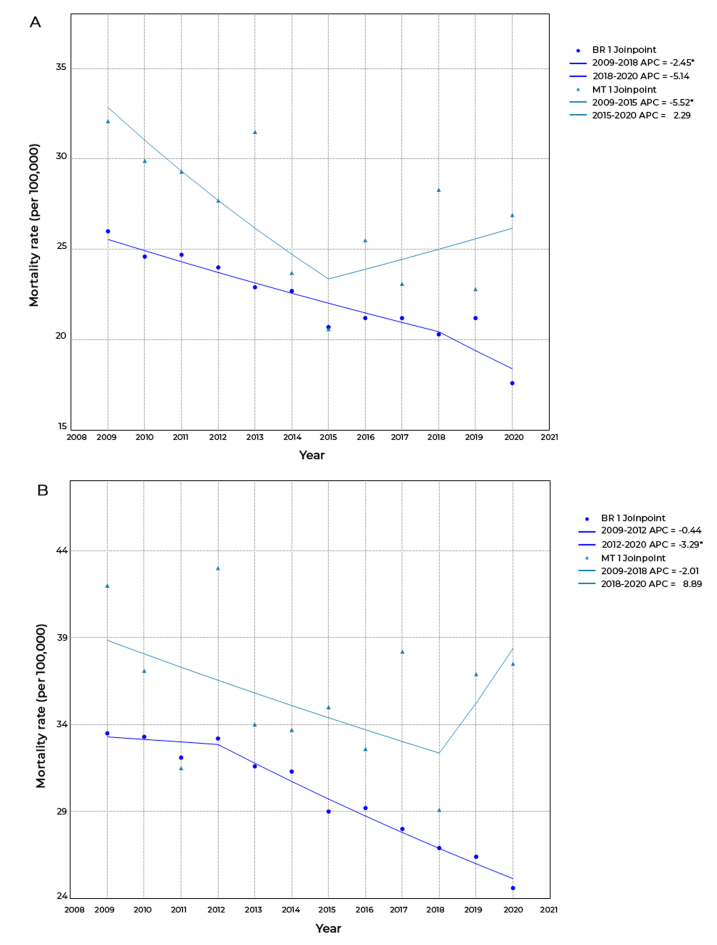
Temporal trend of mortality in the 5-9 age group (A) and the 10-14 age group (B), Brazil and Mato Grosso, 2009-2020

In Brazil as a whole, in the 5-9 years age group, there was a joinpoint in 2018, and the temporal trend was classified as falling (AAPC = -2.9; 95%CI -4.3;-1.6); in the 10-14 years age group, there was a joinpoint in 2012, and the temporal trend was also classified as falling (AAPC = -2.5; 95%CI -3.3;-1.8). In Mato Grosso, this trend proved to be stationary in both age groups (5-9 years, AAPC = -2.0; 95%CI -5.6;1.7; and 10-14 years, AAPC = -0.1; 95%CI -5.9;6.1), joinpoints in 2015, in the 5-9 years age group, and in 2018, in the 10-14 years age group (Table 3). In addition to being higher than the national rates, the Mato Grosso rates showed greater oscillation, compared to expected (Figure 2).

**Table 3:** Average percentage change according to joinpoint regression of the 5-14 age group mortality rates, Brazil and Mato Grosso, 2009-2020

Place	Age group (in years)	Period	APC^a^ (95%CI)	Classification	AAPC^b^ (95%CI)	Classification
Brazil	5 to 9	2009-2018	-2.4^c^ (-3.2;-1.7)	Falling	-2.9^c^ (-4.3;-1.6)	Falling
		2018-2020	-5.1 (-12.8;3.2)			
Mato Grosso		2009-2015	-5.5^c^ (-10.6;-0.2)	Falling	-2.0 (-5.6;1.7)	Stationary
		2015-2020	2.3 (-4.9;10.0)	Stationary		
Brazil	10 to 14	2009-2012	-0.4 (-3.3;2.5)	Stationary	-2.5^c^ (-3.3;-1.8)	Falling
		2012-2020	-3.3^c^ (-3.9;-2.7)	Falling		
Mato Grosso		2009-2018	-2.0 (-5.2;1.3)	Stationary	-0.1 (-5.9;6.1)	Stationary
		2018-2020	8.9 (-24.7;57.4)			

a) Annual percentage change; b) Average annual percentage change; c) Significance test using the Monte Carlo permutation method.

## Discussion

Between 2009 and 2020, mortality in the 5-14 age group in both Brazil and Mato Grosso was predominantly male, preventable and due to external causes, especially traffic accidents and assaults, being higher among those aged 10-14 years compared to those aged 5-9 years. There was a reduction in mortality rates at the end of the period in relation to the values found at the beginning. 

Unlike Brazil, which maintained a falling trend in both age groups, Mato Grosso showed a stationary trend for both groups. Even though this trend is not statistically significant, when considering the high rate of mortality in the state, it raises an alert with regard to change in the state context.

These results are in line with the reduction in mortality of children and adolescents aged 10-14 years in countries with different income levels between 1955 and 2004. In that period, among people under 24 years of age, the 5-14 age group had the lowest number of deaths, and the reduction in mortality among 5-9 years old was greater than among 10-14 years old.^14^

The difference in the speed of decline in the rates, between age groups, continued to be seen worldwide from 1990 to 2016.^2^ ^,^ ^3^ The greater drop in deaths of 5-9 years old can be attributed to the indirect benefit of public policies aimed at children under 5 years of age, while the causes of death for 10-14 years old are more difficult to prevent, due to the greater frequency of deaths from external causes.^4^

The mortality profile of children and adolescents in Mato Grosso was similar to that of other Brazilian states,^16^ ^,^ ^17^ and that of other countries.^18^ ^,^ ^2^ ^,^ ^3^ ^,^ ^5^ Besides external causes, neoplasms, respiratory, neurological and infectious diseases figure in the ranking of causes of death in these age groups, pointing out the crucial role of socioeconomic aspects in the order of occurrence of deaths in each region.^2^ ^,^ ^3^ ^,^ ^5^ ^,^ ^15^ ^,^ ^16^ ^,^ ^17^ ^,^ ^18^ ^,^ ^19^

The reduction in mortality from infectious diseases and cancer may reflect improvements in access to and quality of health care services, such as vaccination, early diagnosis methods, timely use of antibiotics, and appropriate surgical treatment.^19^

The predominance of deaths from external causes, especially those resulting from traffic accidents, is due both to the low frequency of morbidity in this group and also to the greater vulnerability of people who live in high population density areas, with high rates of violence and precarious and unsafe urban and road infrastructure. Added to the natural characteristics of their age (less physical structure, difficulty in identifying risks and dependence on help from others), such factors can be key to the increase of this outcome among children.^20^

In Brazil as a whole, the mortality rate for the main preventable underlying causes was similar among the 5-9 and 10-14 age groups for almost all causes. However, despite the general reduction in mortality from preventable causes since the 2000s, in 2013, the mortality rate due to external causes was significantly higher in the older age group.^16^

In Cuiabá, the capital of Mato Grosso state, in 2009, fatal victims between 5 and 14 years old accounted for 4.6% of deaths from external causes in people under 24 years of age: the majority resulting from accidents, and, with effect from 10 years of age, showing an expressive predominance of males and an increase in victims of assault.^21^

Considering that traffic accidents are mostly predictable and preventable, monitoring these events is an important tool for the implementation of public prevention and health promotion policies.^22^ The implementation of traffic safety policies - such as the use of seatbelts, helmets and car seats, investing in road safety, encouraging safe driving behavior, and criminalizing drug-impaired driving - has been associated with a reduction in child and adolescent traffic fatalities in Brazil and worldwide.^20^ ^,^ ^21^ ^,^ ^22^

In Mato Grosso, drowning is an important cause of death among external causes, especially in children 5-9 years old. The climatic and hydrographic aspects of the state favor aquatic leisure activities, increasing exposure, which requires continuous surveillance, greater attention to safety structures in risk areas, and family behavior changes.^23^

In order of importance, assaults are the third leading cause of death in those aged 5-14 years old, indicating a change in the mortality pattern associated with increasing age.^6^ Studies in Brazil and elsewhere^17^ ^,^ ^20^ ^,^ ^24^ have also revealed that in this age group there is increased risk of death due to assault as age increases, especially among males.

Predominance of male mortality, in both age groups, and a high number of preventable deaths have been found by different studies around the world. In recent decades, there has been a predominance of male mortality in the 5-14 age group in countries with different ethnic and socioeconomic characteristics, such as Ethiopia (52%), India (52%), China (57.1%), Brazil (59.4%) and Mexico (63.4%).^2^ ^,^ ^18^ A similar scenario has been found in different Brazilian states: Minas Gerais (64.3%), Maranhão (53.5%) and Rio Grande do Norte (65%).^25^ ^,^ ^26^ ^,^ ^27^

In general, young males are usually exposed to higher risk situations, such as aggressive behavior, urban violence, drug trafficking, alcohol and other drug use, work activities, and dangerous driving, thus being more associated with the external cause mortality profile.^15^

It was not possible to obtain information on the resident population according to race/skin color in each age group, thereby limiting the analysis of this variable to the description of absolute and relative frequencies only. The categorization used was not sufficient to enable analysis of possible inequities related to the race/skin color of the children and adolescents who died.

Since this study analyzed secondary data, extracted from death certificates, the possibility exists of errors occurring when filling out the original death certificate and inputting the data to the information system. However, the results obtained are in line with those described in the literature and corroborate the need for greater focus on this outcome.^2^ ^,^ ^9^ ^,^ ^10^

Studies of causes of death, carried out by the World Health Organization (WHO), and studies of the global burden of morbidity reveal that, in several countries, the process of collecting, recording, and making mortality data available needs to be improved, with regard to underestimation of deaths and their causes, especially in the 5-14 age group, due to their lower visibility.^2^ Underreporting of deaths interferes with important epidemiological indicators. Improving the quality of socio-demographic and morbidity and mortality information provides input to the decision-making process in public health service management, for the prevention and control of health conditions and problems characteristic of each region.^9^

The evolution of studies and evidence related to mortality in the 5-14 age group allows identification of more appropriate interventions intended to reduce these deaths.^28^ The occurrence of outcomes of this nature implies, in addition to years of potential life lost, epidemiological, social, and economic consequences and, in each affected family, they can leave immeasurable emotional scars. 

The results of the temporal trend analysis may be related to people’s increased social vulnerability. The fiscal austerity measures implemented in Brazil to control the economic crisis that began in 2015 may have negatively impacted social welfare programs and policies, increasing the number of hospitalizations and preventable deaths among children.^29^ The threat to the health and socioeconomic progress achieved so far brings signs of worsening indicators with effect from 2016, as well as the resurgence of diseases that had been eradicated.^11^

The finding regarding the stationary temporal trend, given the high mortality rates identified, with external causes as the main causes of death in children and adolescents, especially traffic accidents, is a cause for alarm and indicates the urgent need for interventions by the various government sectors as well as civil society organizations in the state of Mato Grosso.
